# Repurposing Treatments to Enhance Innate Immunity. Can Statins Improve Neutrophil Functions and Clinical Outcomes in COPD?

**DOI:** 10.3390/jcm5100089

**Published:** 2016-10-11

**Authors:** Georgia M. Walton, James A. Stockley, Diane Griffiths, Charandeep S. Sadhra, Thomas Purvis, Elizabeth Sapey

**Affiliations:** 1 Institute of Inflammation and Ageing, University of Birmingham, Birmingham, B15 2TT, UK; g.m.walton@bham.ac.uk (G.M.W.); charandeepsadhra@hotmail.com (C.S.S.); TXP963@student.bham.ac.uk (T.P.); 2 Lung Function and Sleep, University Hospitals Birmingham NHS Foundation Trust, Birmingham, B15 2TT, UK; james.stockley@uhb.nhs.uk; 3 Respiratory Research, Research and Development, University Hospitals Birmingham NHS Foundation Trust, Birmingham, B15 2TT, UK; diane.griffiths@uhb.nhs.uk

**Keywords:** neutrophil, inflammation, COPD, statins, chemotaxis, simvastatin

## Abstract

Drug classes used in the treatment of Chronic Obstructive Pulmonary Disease (COPD) have not changed for many years, and none to date have shown disease-modifying activity. Statins are used to help reduce cardiovascular risk, which is high in many patients with COPD. Their use has been associated with improvements in some respiratory manifestations of disease and reduction in all-cause mortality, with greatest reductions seen in patients with the highest inflammatory burden. The mechanism for these effects is poorly understood. Neutrophils are key effector cells in COPD, and correlate with disease severity and inflammation. Recent in vitro studies have shown neutrophil functions are dysregulated in COPD and this is thought to contribute both to the destruction of lung parenchyma and to the poor responses seen in infective exacerbations. In this article, we will discuss the potential utility of statins in COPD, with a particular emphasis on their immune-modulatory effects as well as presenting new data regarding the effects of statins on neutrophil function in vitro.

## 1. Introduction

COPD is a chronic inflammatory condition of the lungs associated with significant systemic manifestations and co-morbidities [1]. It is defined by expiratory airflow obstruction that is not fully reversible (by bronchodilation) and during the course of the disease nearly all patients decline functionally, symptomatically and physiologically as well as demonstrating continued progressive lung damage [2]. COPD is heterogeneous and encompasses a number of diseases and syndromes, termed phenotypes due to their impact on clinical outcomes. Recognised phenotypes include emphysema, bronchiolitis, bronchiectasis, chronic bronchitis, bacterial colonisation, and frequent exacerbations (or flares in symptoms often requiring additional short-term treatment with corticosteroids and antibiotics) and patients suffer with differing burdens of each of these disease manifestations.

Of all the co-morbidities associated with COPD, one of the most important is cardiovascular disease (CVD). COPD patients have a higher risk of CVD than people without COPD [3,4], even when shared major risk factors (including smoking, hypertension, diabetes, inactivity, poor diet, and older age) are considered [5,6,7].

COPD is now the third leading cause of death world-wide [8] and is a major cause of morbidity and hospital admissions. In the UK, COPD is estimated to cost the economy £3billion per year, with NHS costs of £900 million annually [9]. In the developed world, cigarette smoking is the most important risk factor for disease and around 80% of COPD patients are current or ex-smokers [10] however there is emerging impact of biomass fuel use on disease prevalence in less developed countries [11,12] and despite the decline in smoking the burden of COPD continues to rise.

There are currently no disease modifying medications in COPD, and the mainstay of treatment remains bronchodilators and generic anti-inflammatory corticosteroids, although there is controversy about their high utility in this disease [13]. There is a pressing need for new treatments, but with the increasing costs of drug discovery, there is renewed emphasis on the repurposing of drugs in different clinical settings to gain maximal benefit from the therapies we already have.

### 1.1. Inflammation, the Neutrophil, and COPD

COPD is associated with an abnormal, chronic, and injurious inflammatory response [14]. A large body of research supports a central role of the neutrophil in these inflammatory processes. Airway neutrophilia is a feature of COPD, shown in all clinical phenotypes including patients with a predominance of emphysema, with chronic bronchitis and bacterial colonisation, frequent exacerbators, and even those with evidence of higher eosinophil activity [15]. The degree of this neutrophilia also correlates with severity of disease and rate of physiological decline [16,17]. Neutrophil proteinases, especially neutrophil elastase, are implicated in all pathological features of COPD in vitro and in animal models [15]; proteinases released by neutrophils are associated with the development of emphysema [18], contribute to destruction of the extracellular matrix [19] and are associated with mucus hypersecretion [20].

Neutrophils are the dominant circulating leukocyte and represent a key component of the innate immune system. In response to infection/inflammation, neutrophils leave the circulation and migrate to sites of infection where they utilise a variety of mechanisms to contain and kill invading pathogens. This includes phagocytosis, degranulation, production of reactive oxygen species (ROS), and release of neutrophil extracellular traps (NETs).

Neutrophils are recruited to areas of inflammation by responding to inflammatory mediators released from inflamed or infected tissue, such as Interleukin 8 (CXCL8) and leukotriene B4 (LTB4). Laminar blood flow through post capillary venules forces heavier leukocytes (including neutrophils) against the endothelial walls. During inflammatory events, endothelial cells upregulate the expression of selectins (P-selectin, E-selectin) and intercellular Adhesion Molecule (ICAM) adhesion molecules on their luminal surfaces [21] which bind P-selectin glycoprotein ligand-1 (PSGL-1), l-selectin and integrins such as CD11a/CD18 on the neutrophil, supporting neutrophil capture and transmigration through endothelium into the tissues [22]. Before neutrophil movement can initiate, the cell must orientate and polarise itself along the gradient formed by increasing inflammatory mediator concentrations by a mechanism known as “compassing” and then form pseudopods that effectively drag the cell in the required direction [23,24]. Pseudopod formation is stimulated by an intracellular process controlled by the localisation of Phosphatidylinositide 3-kinase (PI3K), SH-2 containing inositol 5’-polyphosphatase 1 (SHIP1), and Phosphatase and tensin homolog (PTEN). PI3K converts Phosphatidylinositol 4,5-bisphosphate (PIP_2_) into Phosphatidylinositol (3,4,5)-triphosphate (PIP_3_) which has downstream effectors that induce F-actin polymerisation and protrusion of actin filaments from the neutrophil membrane; thus creating a pseudopod [25]. SHIP1 and PTEN regulate the reverse reaction and reduce the amount of local PIP_3_, preventing actin polymerisation but also deactivating integrins [26].

For a neutrophil to migrate in the correct direction, it must have higher PI3K activation in the area of the cell facing the chemical gradient (leading edge) and higher SHIP1, PTEN activation (to impede PI3K activity) on cell surfaces opposite to the leading edge (the uropod). This creates an anterior posterior PIP_3_ gradient, polarising the neutrophil [27]. The activity of SHIP1, PTEN, and PI3K are regulated by small GTPases. GTPases are a large family of hydrolase enzymes that can bind and hydrolyze guanosine triphosphate. They switch between inactive GDP bound forms and the active GTP bound forms in order to trigger the activation of PI3K and PTEN in their correct cytosolic locations. The activity of the small GTPases is initiated by the binding of chemoattractants such as LTB4 and CXCL8 to the neutrophil surface [28].

Cigarette smoke (CS) contains a multitude of toxic products and is thought to initiate the inflammatory processes within the lungs in COPD in most patients [29]. Initially, epithelial cell damage by CS causes release of damage associated molecular patterns (DAMPs) which interact with pathogen recognition receptors (PRRs) and toll-like receptors (TLRs) on other epithelial cells, resulting in the release of inflammatory cytokines such as CXCL8. The resultant infiltration of inflammatory cells—predominantly neutrophils, but also macrophages and T-cells—into the lung perpetuates further cytokine release, resulting in this chronic inflammatory condition and the degree of the inflammatory response correlates with lung disease severity [30]. As neutrophils migrate through complex tissues, they release ROS and proteinases to facilitate their movement. Alpha-1 antitrypsin (AAT) is an anti-proteinase and inhibits neutrophil elastase on a 1:1 basis, however the interstitial concentration of AAT is significantly lower than that of neutrophil elastase following release from the cell [31] leading to an area of uninhibited elastase activity with subsequent obligate tissue damage [32]. COPD is chronically associated with vast numbers of neutrophils passing through the lungs [33] and so the capacity for tissue damage is considerable and sustained.

Once neutrophils reach their target sites, they have a number of methods to remove bacterial colonies and cell debris in the lung; phagocytosis, degranulation and release of antimicrobial peptides, respiratory burst (release of ROS), and NETosis (release of extracellular meshwork laden with antimicrobial agents).

Phagocytosis is a receptor mediated process whereby the neutrophil binds bacteria through IgG and complement opsonisation or Pathogen Associated Molecular Patterns (PAMPs) [34] before engulfing and containing the bacteria within an internal phagosome. The phagosome fuses with intracellular granules (forming a phagolysosome) and exposes the microbe to various anti-microbial agents and proteins [35]. The respiratory burst is also stimulated by endocytosis of bacteria and results in the exposure of engulfed microbes to toxic oxidative products. ROS production is catalysed by NADPH oxidase which forms on the internal membrane of the phagosome and converts oxygen to toxic hydrogen peroxide and hydroxide radicals [36]. Increased oxidative stress increases airway mucous secretion in animal models through upregulation of the mucin gene, which stimulates differentiation of epithelial cells to goblet cells [37,38,39]. Both ROS release and phagocytosis require small GTPase activity within the cell, which coordinate the required cytoskeletal reorganisation needed to move bacteria and ROS generating constituents into close proximity [40].

NETs are extracellular DNA meshworks laden with azurophillic and specific granule proteases and histones which neutrophils release in some inflammatory environments. The NET effectively captures microbes and provides a platform for bacteriocidal proteinases and histones [41]. NET release is mediated by protein kinase C (PKC), which activates the GTPase Rac2 and forms part of the NADPH oxidase complex. ROS production is increased in the cytosol and perforates the neutrophils own nuclear envelope allowing the granule proteinases to mix with the nuclear DNA and histones to form a NET chromatin lattice. Permeation of the neutrophil membrane then releases the NETs [42].

Initially, there was controversy as to whether the sustained neutrophilic inflammation present in COPD was a ubiquitous response to the inflammatory environment caused by CS (or other toxic environmental exposures), or whether neutrophil functions, and the resultant inflammation, were dysregulated in COPD, and a fundamental component of disease. There is a building body of evidence to support the latter hypothesis. Only a quarter of smokers develop clinically significant COPD [43] and smoking cessation does not resolve neutrophilic inflammation in patients with COPD, in contrast with smoking controls [44,45,46], supporting the presence of other—as yet undefined—susceptibility factors for disease pathogenesis. Furthermore, previous work from our group has shown distinct alterations in the ability of neutrophils from patients with COPD to migrate towards a plethora of chemo-attractants; whereby cells migrate with increased overall speed, but reduced migrational accuracy [47]. Hence, despite being able to move faster, these neutrophils have a reduced ability to move directly towards inflammation or invading bacteria. Recent studies also suggest that following pathogen clearance, a proportion of neutrophils may return to the vasculature [48]. In these circumstances, the obligate area of tissue damage [49] (described earlier) may be increased; due to the more circuitous routes of migration both to and then from the inflamed tissues, both in the lung and systemic vasculature. In keeping with this, endothelial dysfunction is a well-recognised feature of COPD [50] and is in part mediated by neutrophil proteinases and reactive oxygen species [51]. As well as reduced migratory accuracy, neutrophils from COPD have been associated with enhanced respiratory burst responses (both spontaneous production and following stimulating with phorbol myristate acetate (PMA) [52,53]) and one study has implicated neutrophil NETs in COPD (with NET content in sputum correlating with disease severity) [54]. Also, it has been speculated that infiltrating dendritic cells may collect and present some of the high volumes of self-DNA released as NETs, potentially stimulate an autoimmune response in COPD [55], where anti-elastase autoantibodies have been both described and pathologically associated with disease [56]. NETs have also been associated with tissue damage and disease including endothelial dysfunction and atherosclerosis [57,58]. Hence, the neutrophil is increasingly recognised as an immunotherapeutic target in COPD [59] but it is unclear which treatments might positively impact on cellular functions associated with tissue damage without impairing their crucial bactericidal activity.

### 1.2. Cholesterol, Statins, and Cardiovascular Disease

**Figure 1:**
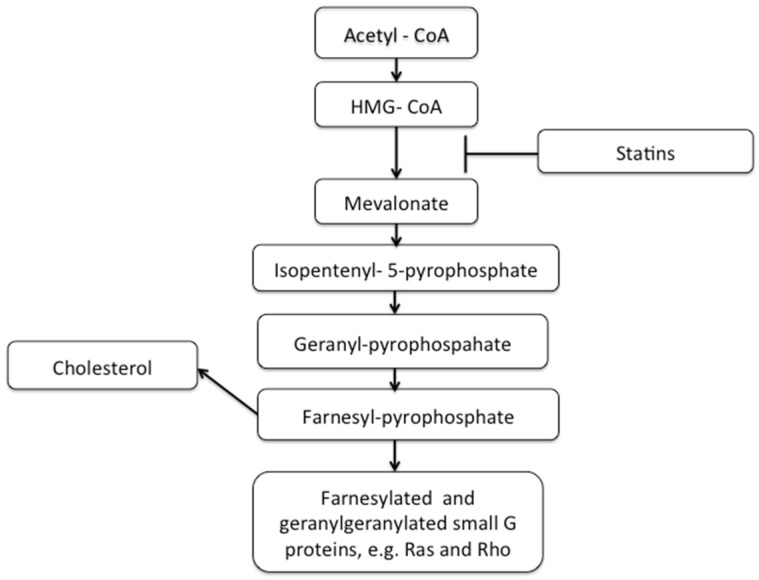
The HMG-CoA reductase pathway. Statins act by competitively inhibiting HMG-CoA reductase, reducing the rate by which HMG Co-A reductase is able to produce mevalonate, the next molecule in the pathway that produces cholesterol. This pathway is also responsible for isoprenoid formation, these are required to modify and activate small GTPases.

3-hydroxy-3-methyl-glutaryl-coenzyme A (HMG CoA) Reductase Inhibitors (commonly referred to as “Statins”) are primarily indicated for the treatment of dyslipidaemia, to reduce cardiovascular risk. The evidence supporting statin-associated CVD risk reduction is extremely robust (for example, [60]) and statins appear safe and well tolerated in many patients [61]. The primary mechanism of action is inhibition of HMG-CoA reductase, an enzyme which catalyses the reaction between HMG-CoA and mevalonate, and forms the rate-limiting step of cholesterol synthesis, thereby statins are able to reduce cholesterol (see Figure 1).

However, evidence has consistently shown that statins reduce all-cause mortality irrespective of baseline low-density lipoprotein (LDL) cholesterol [62,63] with the proportional reduction in major vascular events the same in both low and high risk patients [62]. This, and other research, has led to the currently controversial hypothesis that cholesterol is not causally associated with cardiovascular disease [64], and that the therapeutic effects of statins may reflect their other, pleiotropic mechanisms of action, in particular those which are anti-inflammatory or immunomodulatory.

In addition to reducing cholesterol synthesis, statins also reduce production of all products downstream of HMG-CoA, including inhibition of isoprenoid production. Isoprenoids are utilised by cells for the post-translational modification and activation of small GTP-ases such as Ras and Rho; molecules that are essential for a number of inflammatory cellular functions including adhesion, cell recruitment, and immune cell de-granulation [65,66] (as outlined above). See Table 1 for a summary of the pleiotropic immune-modulatory and anti-inflammatory actions of statins.

There is evidence that inflammation is central to CVD with C-Reactive Protein (CRP) and tumour necrosis factor alpha (TNFα) concentrations predictive of cardiovascular events [80,81]. There is also significant data to support mevalonate pathway activation in CVD. Endothelial dysfunction/plaque formation in CVD is associated with increased adhesion molecule expression via Rho activity and, across classes, statins have been shown to reduce adhesion molecule expression in both immune and endothelial cells via inhibition of GTPases [82,83]. Atherosclerotic plaques are characterised by immune cell infiltration and statins have been shown to reduce this both via the reduction of plaque/endothelial derived chemoattractants and by altering immune cell function to reduce pro-inflammatory activity [84]. Plaque formation has been associated with macrophage and T cell infiltration, increased MMP-2 expression (a macrophage derived proteinase) and collagen proteolysis, while statins reduce the cellular content of plaques, increase tissue inhibitor of MMP-1 (an anti-proteinase) and are associated with a higher collagen content, suggesting a stabilizing effect of atherosclerotic lesions by reducing proteolysis [85].

Neutrophilic inflammation is a central component of atherosclerosis [86,87] and there is an increasing interest in the neutrophil’s role in CVD. Neutrophil numbers are associated with impaired microvascular perfusion, left ventricular dilation and adverse cardiac events in patients treated for myocardial infarction [88]. Furthermore, myeloperoxidase (a marker of neutrophil activity) is increased acutely following myocardial damage and predicts CVD outcomes [88,89]. Neutrophils promote clot formation through interactions with platelets, proteolytic cleavage of clotting factors, and release of prothrombotic NETs [90]. Neutrophils promote atherosclerosis and plaque rupture by enhancing monocyte and further neutrophil infiltration, producing oxidized low density lipoprotein (oxLDL), and releasing proteolytic enzymes that degrade the fibrous cap of the plaque [91]. Statins are known to impact on many of these inflammatory processes. Studies have described reduced adhesion of neutrophils to fibronectin with 10 µm simvastatin and a decline in activated expression of CD11b [92] and demonstrated that statins are able to reduce degranulation [93], migration [94], and netosis [95] during inflammatory events.

**Figure 2:**
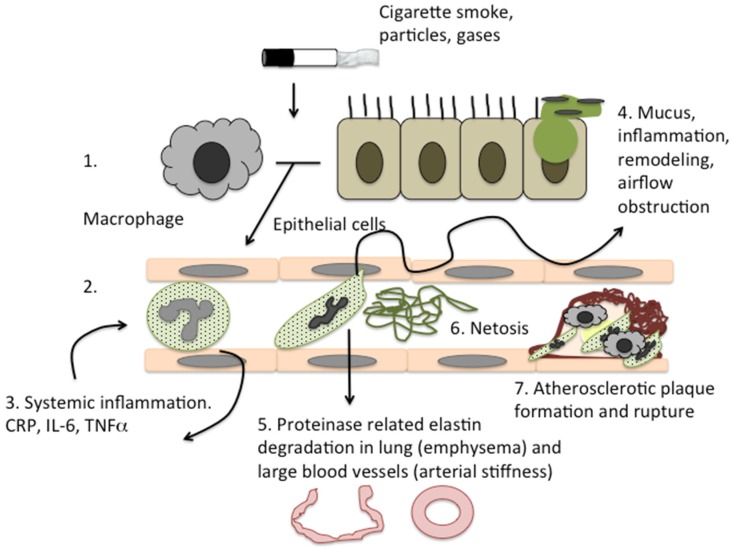
Shared inflammatory mechanisms in COPD and CVD. Chronic cigarette smoke exposure or other toxic inhalants in susceptible people leads to (**1**) the sustained activation of airway macrophages and epithelial cells; (**2**) The release of inflammatory cytokines causes endothelial activation and neutrophils recruitment to the airways, however COPD poor migratory accuracy and increased reactive oxygen species release leads to (**3**) increased systemic inflammation. This activates endothelial cells further, recruiting more neutrophils and as well as causing (**4**) bystander tissue damage to both blood vessels and the lung parenchyma; (**5**) Neutrophil proteinases degrade elastin fibres both in the lung and large blood vessels, causing emphysema and arterial stiffness as well as increasing the activity of macrophage proteinases such as MMP12. Neutrophil netosis (**6**) adds to the inflammatory burden, and promotes platelet aggregation in small vessels due to endothelial damage (**7**). Atherosclerotic plaques form over areas of endothelial damage, which are infiltrated by immune cells including neutrophils during plaque rupture.

Many of the inflammatory processes associated with CVD (and described above) have also been described in the pathology of COPD, where pulmonary and systemic inflammation is also significantly raised [33]. In light of this, it has been hypothesised that lung damage in COPD and cardiovascular events may share a common inflammatory pathology, with the severity of one predicting the onset of the other [96]. See Figure 2 for a schematic of how neutrophilic inflammation may be implicated both in COPD and CVD. This has potential importance, as disease-modifying drugs that target inflammation in COPD may reduce cardiovascular events, and treatments that reduce cardiovascular morbidity may impact on COPD progression in this high-risk population. Since statins have been shown to modify inflammatory processes implicated in both diseases, there are theoretical reasons to consider their use in COPD, not just to modify cardiovascular risk but also to treat manifestations of lung disease.

### 1.3. Statins and Clinical Outcomes in COPD

There are an increasing number of studies examining the potential impact of statin use in COPD. Observational studies of COPD have described a slower decline in pulmonary function in patients taking statins [97,98]. Furthermore, a number of retrospective, observational studies have reported statin-associated reductions in death from infection (pneumonia and influenza) [99], all-cause mortality [100] and a recent meta-analysis of cohort studies detected a protective effect of statin treatment on the risk of COPD exacerbation with or without hospitalization [101]. In animal models, statins have been shown to be protective against the development of emphysema, inhibiting lung parenchymal destruction and the development of pulmonary hypertension. In the same study, statins also inhibited peribronchial and perivascular inflammatory cell infiltration in lung tissue [102]. In human studies, statins have been shown to reduce endothelial dysfunction and systemic inflammation [103].

However, these results have not been universally replicated and some studies have shown no benefit [104] including a recent meta-analysis of randomised controlled trials in COPD [105]. The discordance between observational studies, in vitro studies, animal models (which generally show benefit), and interventional trials (which often do not) mirrors the situation seen for research into statin use in sepsis [106] and the reason for the disparity may reflect experimental design. Observational studies usually include older patients who have been taking statins for many months, in vitro cellular studies often utilise high concentrations of statins (which sometimes exceed standard prescribing regimes by over 10-fold), while clinical trials have often used low dose statins in patients of varying age as an acute intervention. Furthermore, clinical trials have utilised statins for short periods of time. Some patients experience side effects with statin use including (albeit rarely) potentially serious adverse events such as rhabdomyolysis and hepatitis, and these must be carefully considered when instigating a potentially long term treatment. Statins may be helpful adjuvants to treat inflammation in COPD, but the questions are: which statin and at what dose? Which patients? When should treatment start and for how long for?

All statins studied have shown some anti-inflammatory outputs in keeping with their shared mechanism of action and therefore the choice of statin does not appear important in studies reported to date. Current evidence points to a high dose being associated with improved inflammatory outcomes. Simvastatin (5–20 mg/kg) used with inhalation injury in mice was effective in attenuating lung damage but only at the higher dose [107]. In a double-blind placebo-controlled study, intravenous endotoxin produced a detectable systemic inflammatory response which was prevented by pre-emptive dosing with 80 mg Simvastatin in humans [108] but not at a lower dose.

The patient group under study is also likely to be important. The recently reported STATCOPE study randomised COPD patients to receive 40 mg Simvastatin to determine impact on exacerbation rates. Inclusion criteria included a diagnosis of COPD and the use of supplemental oxygen, receipt of systemic glucocorticoids or antibiotic agents for respiratory problems, or presentation to the emergency department or hospitalization for COPD exacerbation in the preceding year [109] but frequently exacerbating patients (defined as two or more per year) were not specifically selected. This (and the lower dose of 40 mg simvastatin) may have potentially impacted on findings.

Finally, the endpoint is likely to be crucial. It is unlikely that statins will act as a panacea for all manifestations of COPD or all inflammatory processes. Dysregulated neutrophil functions have been associated with tissue damage in COPD, particularly functions associated with increased or indiscriminate neutrophil elastase activity, which in turn has been associated with emphysema. If neutrophils are central to the development of COPD, one would expect a disease-modifying drug to impact positively on these functions, and there is evidence to support this. In a small controlled trial, statin therapy was associated with a decrease in sputum leptin, absolute neutrophil counts, and the incidence of COPD exacerbations [110].

## 2. Statins and Neutrophil Migration in COPD; In Vitro Studies

Previously, we identified increased migratory speed of movement in any direction (termed chemokinesis) but reduced migratory accuracy towards inflammation (termed chemotaxis) in systemic neutrophils isolated from patients with COPD and related this to systemic neutrophil elastase activity and the potential for tissue damage [47]. We have studied the effects of therapeutically relevant concentrations of Simvastatin on neutrophil migratory dynamics in both patients with COPD (average age 69.5 years ± 8.5) and age-matched healthy control subjects (average age 64.4 years ± 12). Chemotaxis was assessed in neutrophils isolated from whole blood using an Insall Chamber (Weber Scientific International Ltd., Teddington, UK) and time-lapse video microscopy, as described previously [111]. Neutrophils were migrated towards 100 nm CXCL8 (R & D Systems, Abingdon, UK) or 10 nm fMLP (Sigma-aldrich, Dorset, UK) for 12 min, parameters which were optimised in a series of validation experiments. Cell migration was imaged using a Leica DM6000 B microscope fitted with a DFC 360FX monochrome digital camera and migratory paths tracked using the Java software, ImageJ (Wayne Rasband, NIH, Bethesda, MD, USA).

**Figure 3:**
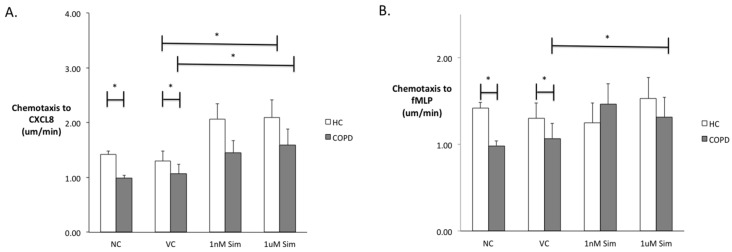

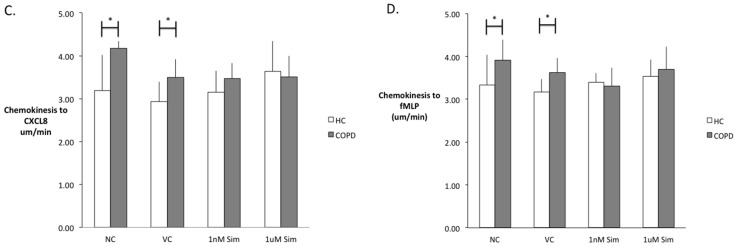
Neutrophil migratory accuracy is improved in COPD following in vitro exposure to simvastatin. Neutrophil migratory chemotaxis (**A**,**B**) (accuracy of movement towards a chemoattractant) and chemokinesis (**C**,**D**) (random speed of movement in any direction) from *n* = 8 patients with COPD and *n* = 10 age-matched healthy controls towards 100 nm CXCL8 (**A**,**C**) or 10 nm fMLP (**B**,**D**) were compared. White bars represent healthy controls (HC) and gray bars represent patients with COPD. “NC” is migration towards CXCL8 or fMLP when neutrophils were incubated with buffer alone, “VC” is migration towards the chemoattractants when neutrophils were incubated in vehicle control. “Sim” is migration towards the chemoattractants when neutrophils were incubated with simvastatin (concentrations as shown). Data presented as mean and standard error of the mean. * represents *p* value < 0.05. Data was compared using Mann-Whitney-U or Kruskal Wallis tests. Neutrophils from patients with COPD were faster than those from healthy controls, but less accurate when migrating towards fMLP and CXCL8. Simvastatin improved accuracy of migration (chemotaxis) towards CXCL8 and fMLP in patients with COPD, and towards healthy controls towards CXCL8. Simvastatin did not affect the speed of migration in either group.

Neutrophils were incubated with Simvastatin sodium salt (Merck Millipore, Nottingham, UK) at 1 nm or 1 µm or vehicle control (dimethyl sulfoxide) or buffer (negative control) for 40 min prior to migration studies and statin concentrations were chosen to represent known steady-state plasma concentrations of Simvastatin from 10 mg to 80 mg [112]. Appropriate incubation time was chosen following appropriate time-course experiments. Of note, the same defect (increased chemokinesis but reduced chemotaxis) was seen in COPD neutrophils compared to those from healthy controls in untreated neutrophils, as previously described. Forty minutes of in vitro treatment of isolated peripheral blood neutrophils with either 1 nm or 1 µm simvastatin was able to improve neutrophil velocity towards both CXCL8 and fMLP in patients with COPD, restoring migratory accuracy to levels seen in age-matched healthy control neutrophils. See Figure 3.

These results are in keeping with other in vitro assessments of the ability of statins to impact upon pro-inflammatory events. Importantly in the studies described above, simvastatin did not inhibit the ability of the cell to respond to infection/inflammation but rather improved it, however there have not been studies of statin use and NETosis, phagocytosis, or ROS release in neutrophils from COPD patients, and these would be needed to further assess the likely impact of statins on crucial neutrophil functions. Evidence from other in vitro human studies have described potential impairment of neutrophil bactericidal functions, but these have used non-therapeutic concentrations (such as 10–50 µm rather than the therapeutic 1 µm [113]) making interpretation difficult. In contrast, mouse studies utilising more relevant doses of drug have described reductions in NET formation in murine models with improved responses to inflammatory challenges [95]. In summary, these early studies by ourselves and others have suggested statins may impact positively on some neutrophil functions thought central to the pathogenesis of both COPD and CVD, but further investigation is required.

## 3. Conclusions

COPD remains a significant cause of mortality and morbidity globally, and our therapeutic tools for reducing its impact are limited. There is clear evidence of improved CVD morbidity and mortality in COPD following statin use, but the shared inflammatory mechanisms that have been described in both COPD and CVD provide theoretical reasons to support the use of statins in COPD progression as well. Statins have been associated with improved lung outcomes in in vitro experiments, in animal models, and in observational studies of COPD. However, results of randomised controlled trials have shown limited efficacy on COPD-related outcomes such as exacerbation frequency. Disparity in COPD-related results may reflect experimental design and the patients and outcomes that were selected. The persistent signal reported in many studies has maintained interest in these drugs for this debilitating disease. Further in vitro studies and proof of principle clinical trials using statins at high doses—with cellular as well as clinical outcomes—in carefully selected patients may provide more insight into their potential utility.

## Figures and Tables

**Table 1:** The anti-inflammatory and immunomodulatory effects of statins.

Effect	Model/Cell-Type	Reference
**Increased NF-IκB expression**	Human hepatocytes	[67]
	Human endothelial cells	[68]
**Phosphorylation of Akt**	Human endothelial cells	[69]
**Decreased TNFα production**	Human volunteers	[70]
**Decreased IL-6 production**	Human volunteers	[70]
	Mice	[71]
**Increased IL-12 production**	Mouse peritoneal macrophages	[72]
**Reduced plasma C-reactive protein**	Human volunteers	[70]
	Human hepatocytes	[67]
**Reduced expression of P-Selectin**	Rat mesenteric endothelium	[73]
**Reduced superoxide production**	Human monocytes	[74]
**Inhibition of matrix metalloproteinase production**	Human lung fibroblasts	[75]
**Regulation of Th1/Th2 polarisation**	Mice	[76]
**Block of LFA-1 ICAM interaction**	Various: isolated proteins, cell lines, murine model	[77]
**Inhibition of MHC expression**	Human macrophages & endothelial cells	[78]
**Inhibition of monocyte chemotactic protein-1 synthesis**	Human peripheral blood mononuclear cells	[79]
